# Association between serum 25-hydroxyvitamin D concentration and the risk of colorectal cancer: A cross-sectional study

**DOI:** 10.1371/journal.pone.0320335

**Published:** 2025-03-25

**Authors:** Yuru Wang, Siqi Feng, Jian Chen, Yun Li, Miaomiao Wang, Tingting Wu, Shujuan Fu, Zhangjie Zhou, Cunya Li, Pantong Wu, Zhiying Wang, Zhixian Zhong, Yi Zhong

**Affiliations:** 1 Shanghai TCM-integrated Hospital, Shanghai University of TCM, Shanghai, China; 2 Shanghai University of Traditional Chinese Medicine, Shanghai, China; 3 Changhai Community Health Service Center, Yangpu District, Shanghai, China,; 4 Tongji University, Shanghai, China; Tehran University of Medical Sciences Endocrinology and Metabolism Research Institute, IRAN, ISLAMIC REPUBLIC OF

## Abstract

**Background:**

The role of vitamin D in the prevention of colorectal cancer (CRC) has been the focus of research, but the results of relevant studies are not entirely consistent. While most studies indicate that vitamin D has a protective effect against CRC, there are also research reports stating that at high serum levels, there is no significant association between vitamin D and CRC, or even an increased risk. Additionally, there are still differences in the recommended serum 25-hydroxyvitamin D [25(OH)D] concentrations among various guidelines or committees. This study examined the association between serum 25-hydroxyvitamin D concentrations and the risk of CRC in US adults.

**Methods:**

This study included 43,678 adult participants from the National Health and Nutrition Examination Survey (NHANES) 2001–2018, and logistic regression modelling was used to examine the association between serum 25(OH)D concentrations and the risk of CRC. We grouped participants according to the classification criteria of the various guidelines available for vitamin D, and controlled for confounding using a multi-model strategy, adjusting for key covariates such as gender, age, race, education level, marital status, family income to poverty ratio (PIR), body mass index (BMI), smoking habits, drinking habits, diabetes, hypertension, dyslipidemia, calcium intake, and total folate intake. We also performed trend tests to evaluate the linear relationship between serum 25(OH)D concentrations and CRC risk, used restricted cubic spline (RCS) plots to assess the dose-response relationship, and conducted further subgroup analyses with interaction tests to examine potential variations in the association across different population groups. We focused on the association between serum 25(OH)D concentration ≤ 75 nmol/L and CRC, again using multivariable logistic regression with a multi-model strategy and RCS plots.

**Results:**

A total of 43,382 participants without CRC and 296 participants with CRC were included in this study. In the fully adjusted model, participants with serum 25(OH)D < 50 nmol/L had more than twice the risk of developing CRC compared to those with levels of 50–< 75 nmol/L (<30 nmol/L: Odds Ratio [OR] = 2.038, 95% Confidence Interval [CI]: 1.011–4.109; 30– < 50 nmol/L: OR = 2.090, 95% CI: 1.361–3.211). The negative correlation between serum 25(OH)D concentration and the risk of CRC was significant when serum 25(OH)D concentration was ≤ 75 nmol/L (*P *< 0.001). Each 1 nmol/L increase in serum 25(OH)D concentration was associated with an approximately 2.3% reduction in the risk of CRC (95% CI: 0.964–0.990).

**Conclusions:**

Our findings indicate a strong inverse association between serum 25(OH)D concentrations and the risk of CRC, particularly when levels are ≤75 nmol/L. Maintaining serum 25(OH)D above 75 nmol/L is associated with a lower CRC risk and may serve as a cost-effective preventive strategy. Public health measures, including routine vitamin D screening in high-risk populations and targeted supplementation, could further support CRC prevention efforts.

## Introduction

Colorectal cancer (CRC), the third most common malignant tumor in the world, added about 1.9 million new cases worldwide, according to the World Health Organization (WHO) in 2020. The incidence of CRC is expected to increase by 63 per cent by 2040 [1]. The incidence of CRC has continued to rise in many countries as the population ages and the birth rate declines. The development of CRC is a long-term and complex process influenced by a variety of factors, including age, diet, lifestyle, personal medical history and family history, which alter the internal and external environment of the organism and may increase the risk of developing and progressing CRC [2]. Therefore, research into ways to reduce the risk of CRC and to improve the understanding of CRC prevention factors has become an important public health issue.

Vitamin D is an essential fat-soluble vitamin that plays a crucial role in maintaining bone health [3], muscle function [4] and immune regulation [5]. In recent years, increasing attention has been paid to the relationship between vitamin D levels and tumor development and survival. Since 1980, when vitamin D was first suggested as a potential preventive factor for CRC, numerous studies have been conducted across various levels and perspectives. Epidemiological studies generally support an association between low vitamin D levels and an increased risk of CRC [6–8]. A review incorporating four observational studies demonstrates that higher 25-hydroxyvitamin D [25(OH)D] levels are associated with a protective effect against CRC incidence, regardless of whether the analysis compares the lowest and highest categories of 25(OH)D levels or examines a dose-response relationship [9]. Meta-analyses have also reported a significant inverse association between serum 25(OH)D concentration and the risk of CRC. For example, one study shows that each 25 nmol/L increase in serum 25(OH)D concentration is associated with a reduction in the risk of CRC of about 11% (95% CI: 0.81–0.98) [10]. Another mixed-effects dose-response meta-analysis shows a 6% (95% CI: 3%–9%) reduction in the risk of CRC for every 10 nmol/L increase in blood 25(OH)D concentration [11]. However, not all studies have confirmed a significant protective effect. Some research has reported no clear association between serum 25(OH)D levels and overall CRC risk. For instance, a prospective cohort study from the Physicians’ Health Study found no statistically significant link between plasma 25(OH)D concentrations and CRC incidence. While an inverse association was suggested for rectal cancer with a potential threshold effect, no such trend was observed for colon cancer [12]. A nested case-control study within the Multiethnic Cohort found that lower serum 25(OH)D levels were associated with a higher risk of CRC. However, the odds ratios for the higher quintiles, compared to the lowest quintile, remained fairly consistent, showing a 37% to 46% risk reduction, suggesting that the association did not follow a strict dose-response pattern [13]. These inconsistencies suggest that the relationship between vitamin D and CRC risk may be influenced by additional factors, such as study design, population characteristics, and potential differences in vitamin D metabolism. Further research is needed to clarify these discrepancies and determine the optimal serum 25(OH)D concentration for CRC prevention.

Various authoritative bodies establish guidelines and recommendations for the minimum threshold and optimal serum levels of 25(OH)D. While there is a broad consensus that serum 25(OH)D levels should not fall below 25 nmol/L, there exists notable variability in the recommendations regarding the ideal serum concentration of 25(OH)D across different institutions and guidelines [14]. For example, the Institute of Medicine (IOM) recommends that a serum 25(OH)D level of 50 nmol/L is sufficient for bone health in most people [15], while some experts and organisations suggest that 75 nmol/L may be beneficial for general health [16]. There are even some recommendations that are more extreme, such as the 25 nmol/L suggested by the UK Scientific Advisory Committee on Nutrition [17], and the other extreme: 100 nmol/L [18]. In addition, vitamin D deficiency and insufficiency are prevalent worldwide [19], further emphasizing the importance of determining optimal vitamin D levels.

This study aims to determine the serum 25(OH)D concentration associated with a reduced risk of CRC and to address discrepancies in current guideline recommendations on vitamin D levels for CRC prevention.

## Methods

### Data source

The NHANES is a comprehensive research program that employs a combination of interviews and physical examinations to gain insights into the nutritional status and health-related issues of the U.S. population [20]. The biennial survey produced nationally representative sample data, thereby providing the public with a valuable health information resource. All participants provided written informed consent, and each survey cycle is reviewed by the National Center for Health Statistics (NCHS) Ethics Review Board [21]. NHANES employed a multi-stage probability sampling design and weighting, enabling the conduct of cross-sectional surveys without the necessity of further ethical approval. The findings of this research program are of great consequence in determining the prevalence of various diseases and their associated risk factors, offering vital information for the assessment of nutritional status and its role in the promotion of health and the prevention of disease.

### Study participants

**Fig 1:**
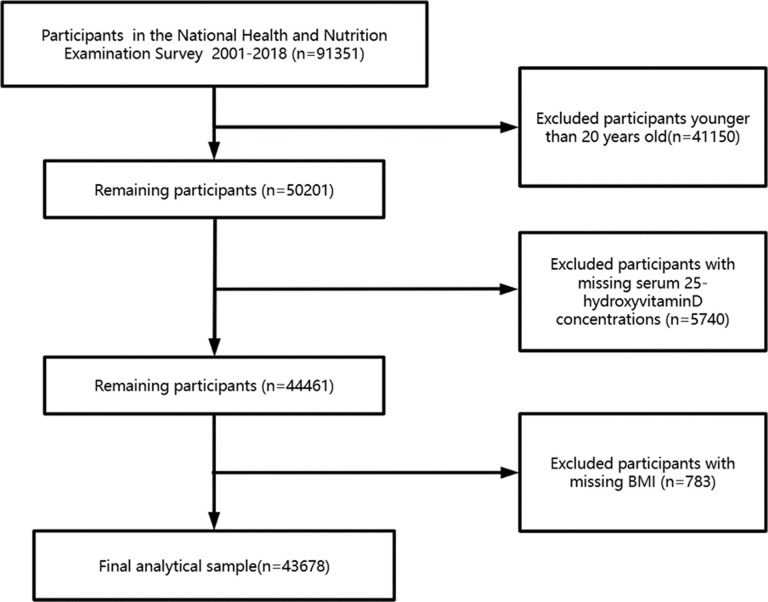
Research flowchart.

In this study, data from nine cycles of NHANES, conducted between 2001 and 2018, were consolidated. The study population comprised individuals aged 20 years and older. Following the exclusion of participants with missing data on 25-hydroxyvitamin D and Body Mass Index (BMI), a total of 43,678 participants were included in the final analysis (Fig 1).

### Acquisition of serum 25(OH)D concentrations

The serum 25(OH)D concentrations were derived from Laboratory data collected through Mobile Examination Centers [21]. Serum 25(OH)D concentrations from 2010 to 2018 were used to quantify 25-hydroxyvitamin D3 and 25-hydroxyvitamin D2 in human serum using ultra-high-performance liquid chromatography-tandem mass spectrometry. The data stated that Total 25-Hydroxyvitamin D is the sum of 25-hydroxyvitamin D3 and 25-hydroxyvitamin D2. The analytes were chromatographically separated on a pentafluorophenyl column with a mobile phase of 69% to 72% aqueous methanol. The NHANES 2007–2010 data for 25(OH)D used a standardized liquid chromatography-tandem mass spectrometry method. Serum 25(OH)D data from 2001 to 2006 had been regression-transformed to equivalent 25(OH)D measurements by liquid chromatography-tandem mass spectrometry. The NHANES project ensured the comparability of data from different years through standardization and regression-transformation methods.

### Access to outcome data for the risk of CRC

The Medical Conditions section of NHANES provides self-reported health status information in the form of questionnaire data. A patient’s CRC diagnosis was categorized based on the following questions: 1. “Age when colon cancer first diagnosed?” (2001–2016); 2. “Age when rectal cancer first diagnosed?” (2001–2016); 3. “cancer - what kind was it?” (2017–2018).

### Assessment and selection of covariates

In this study, we selected covariates based on their established or potential associations with the risk of CRC, as reported in previous literature [2,13,22]. The covariates included gender (male, female), age (20–40 years, 41–60 years, and above 60 years), race (Mexican American, Non-Hispanic White, Non-Hispanic Black, Other), education level (below high school, high school or above), marital status (Yes, No), family income to poverty ratio (PIR) (poor, not poor), BMI (normal weight < 30 kg/m^2^, overweight ≥ 30 kg/m^2^), smoking habits (current, former, never), drinking habits (heavy, moderate, none), diabetes (yes, no), hypertension (yes, no), dyslipidemia (yes, no), calcium intake, and total folate intake. Several epidemiological studies have identified demographic characteristics, socioeconomic status, BMI, lifestyle factors (smoking and alcohol consumption), and comorbid conditions (diabetes, hypertension, and dyslipidemia) as significant predictors of CRC risk. Additionally, dietary factors such as calcium and folate intake are known to influence CRC risk by modulating pathways involved in cell proliferation and DNA repair [2]. The NHANES Sample Person Demographics File provided selected demographic variables such as gender, age, race, education level, marital status, and PIR. A PIR value greater than or equal to 1.00 indicated non-poverty status. BMI was directly provided from the ‘Body Measures’ in the Examination Data, expressed as weight in kilograms divided by height in meters squared (kg/m^2^), and was used to classify weight status. Smoking habits and drinking habits were derived from the Questionnaire Data. Diabetes history was assessed by combining Laboratory Data (glycated hemoglobin, fasting blood glucose, and postprandial blood glucose levels) with Questionnaire Data (insulin use, hypoglycemic medication use, and physician-diagnosed diabetes). Hypertension history was determined by averaging diastolic and systolic blood pressure measurements from Examination Data and incorporating physician-diagnosed hypertension from the questionnaire. Dyslipidemia history was determined based on Laboratory Data of total cholesterol, triglycerides, high-density lipoprotein, and low-density lipoprotein cholesterol levels, as well as communication between patients and healthcare providers from the questionnaire. Intake of calcium and folic acid was calculated from Dietary Data by averaging the intake over two days. If data for one day was missing, the intake from the other day was used instead.

### Statistical analysis

The R programming language (version 4.3.2) and the R Studio integrated development environment (version 2023.12.1) were employed for the extraction, merging, analysis, and plotting of the data. All statistical analyses were weighted in accordance with the principle of the smallest subsample. Continuous variables with a normal or approximately normal distribution are presented as the mean ± standard deviation [mean (SD)], while those with a skewed distribution are shown as the median [interquartile range]. Additionally, weighted counts and percentages for categorical variables are provided. The characteristics of continuous and categorical variables were analyzed using t-tests, chi-square tests, and non-parametric tests, respectively. In accordance with the classification recommendations for 25(OH)D set forth in various guidelines, the population was divided into five groups. The relationship between each group’s 25(OH)D and the risk of CRC was analyzed using a multivariate logistic regression model, and multicollinearity was assessed using the Variance Inflation Factor (VIF). The dose-response relationship was assessed with RCS. Furthermore, subgroup analyses and interaction tests were conducted to evaluate potential interactions. To examine the relationship between 25(OH)D concentrations of ≤75 nmol/L and the risk of CRC, we conducted additional multivariate logistic regression analyses and RCS. The mode imputation of existing cases was used to input missing values for categorical variables, while the median imputation was used for continuous variables. A *P*-value of less than 0.05 was considered statistically significant, while a *P*-value of less than 0.01 indicated a highly statistically significant result.

## Results

### Baseline characteristics of participants

A total of 43,678 participants were included in this study, with an average age of (47.56 ± 17.12) years. There were 21,058 male participants (48.1%) and 22,620 female participants (51.9%), with 296 individuals diagnosed with CRC. Non-Hispanic White individuals accounted for 68.6% of the sample, 63.4% of the participants were married or cohabitating, 83.5% had a high school education or higher, and 87.0% were free from financial stress. The prevalence of dyslipidemia among participants was 46.4%. A higher proportion of CRC patients was over the age of sixty. Table 1 provides a detailed baseline description of the participants. Statistically significant differences in age, race, marital status, education, smoking, history of diabetes, history of hypertension, dyslipidemia, and dietary calcium intake were observed between individuals with CRC and those without CRC.

**Table 1:** Baseline characteristics.

Variables	Level	Total	Non colorectal cancer	Colorectal cancer	*P* value
**Number**		43678	43382	296	
**Gender, n (%)**	Male	21058 (48.08)	20914 (48.11)	144 (43.63)	0.224
	Female	22620 (51.92)	22468 (51.89)	152 (56.37)	
**Age, years (%)**		47.56 ± 17.121			<0.001
	20–40	15749 (38.45)	15744 (38.64)	5 (2.51)	
	41–60	14217 (36.68)	14176 (36.78)	41 (16.61)	
	>60	13712 (24.9)	13462 (24.57)	250 (80.88)	
**Race, n (%)**	Mexican American	7351 (8.01)	7332 (8.04)	19 (2.88)	<0.001
	Non-Hispanic White	19440 (68. 56)	19245 (68.48)	195 (84.54)	
	Non-Hispanic Black	8910 (10.94)	8856 (10.96)	54 (7.68)	
	Other	7977 (12.49)	7949 (12.53)	28 (4.96)	
**Marital, n (%)**	Yes	26493 (63.43)	26345 (63.49)	148 (52.21)	0.001
	No	17185 (36.57)	17037 (36.51)	148 (47.79)	
**Education,**	Below high school	11222 (16.49)	11132 (16.46)	90 (21.03)	0.045
**N (%)**	High school or above	32456 (83.51)	32250 (83.54)	206 (78.97)	
**PIR, n (%)**	Poor	8143 (12.96)	8097 (12.96)	46 (12.44)	0.825
	Not poor	35535 (87.04)	35285 (87.04)	250 (87.56)	
**BMI, kg/m**^**2**^ **(%)**		28.837 ± 6.775			
	<30	16129 (35.93)	16012 (35.92)	117 (36.89)	0.796
	≥30	27549 (64.07)	27370 (64.08)	179 (63.11)	
**Smoking habits,**	now	9060 (21.10)	9023 (21.15)	37 (12.69)	<0.001
**n (%)**	history	10763 (24.96)	10624 (24.86)	139 (44.50)	
	No	23855 (53.93)	23735 (53.99)	120 (42.82)	
**Drinking habits,**	Heavy	5766 (13.04)	5712 (13.03)	54 (15.22)	0.500
**N (%)**	Moderation	28839 (69.29)	28651 (69.29)	188 (69.78)	
	No	9073 (17.67)	9019 (17.68)	54 (14.99)	
**Diabetes, n (%)**	Yes	7199 (12.49)	7102 (12.41)	97 (27.70)	<0.001
	No	36970 (87.51)	36280 (87.59)	199 (72.30)	
**Hypertension,**	Yes	18286 (37.71)	18063 (37.54)	223 (70.83)	<0.001
**n (%)**	No	25392 (62.29)	25319 (62.46)	73 (29.17)	
**Dyslipidemia,**	Yes	20688 (46.44)	20501 (46.35)	187 (63.32)	<0.001
**N (%)**	No	22990 (53.56)	22881 (53.65)	109 (36.68)	
**Calcium (mcg) (median [IQR])**		810.5 [599,1152.5]	810.5 [599,1153]	799 [561,1039.5]	0.041
**Total folate (mg) (median [IQR])**		350.5 [266,490]	350.5 [266,490]	334 [243,468]	0.112
**25(OH)D (nmol/L) (mean±SD)**		67.77 ± 27.184	67.77 ± 27.179	68.11 ± 28.174	0.874

OR, odds ratio; CI, confidence interval; PIR, Poverty income ratio; BMI, body mass index; 25(OH)D, 25-hydroxyvitamin D.

### Association between serum 25(OH)D concentrations and the risk of CRC

The correlation analysis between serum 25(OH)D concentrations and the risk of CRC was conducted using multivariate logistic regression with stepwise adjustment (Table 2). Our findings indicated that serum 25(OH)D concentrations in the range of 30–<50 nmol/L exhibited statistically significant association with the risk of CRC in all adjusted models compared to the reference category of 50–<75 nmol/L (*P* < 0.01). In model 2 (<30 nmol/L, OR = 2.046, 95% CI: 1.011–4.141, P = 0.047; 30– < 50 nmol/L, OR = 2.105, 95% CI: 1.373–3.228, P < 0.001) and model 3 (<30 nmol/L, OR = 2.038, 95% CI: 1.011–4.109, P = 0.047; 30– < 50 nmol/L, OR = 2.090, 95% CI: 1.361–3.211, P < 0.001), the association was significant for lower serum 25(OH)D concentrations. This suggested that individuals with insufficient serum 25(OH)D concentration were at a significantly higher risk of developing CRC. Public health measures, such as dietary supplementation or controlled sunlight exposure, may potentially reduce CRC incidence by maintaining adequate serum 25(OH)D concentration. Additionally, serum 25(OH)D concentrations in the range of 75–<100 nmol/L showed a significant association in the crude model (Odds Ratio [OR] = 1.648, 95% Confidence Interval (CI): 1.106–2.456, *P* = 0.014). However, this association was attenuated after adjusting for potential confounders, suggesting that the crude association may be influenced by other factors. The *P*-value for trend was < 0.05 in Model 1, Model 2 and Model 3, indicating that a linear trend existed between 25(OH)D and the risk of CRC in the five subgroups of 25(OH)D, with a statistically significant trend towards a lower risk of CRC with increasing serum 25(OH)D concentration. These findings imply that continuous efforts to increase serum 25(OH)D concentration could have a protective effect against CRC. Multicollinearity was not present for all variables (variance inflation factor, VIF < 5) (Table 3).

**Table 2:** Association between 25(OH)D and the risk of CRC in participants.

Variable	25(OH)D (nmol/L)^a^					*P* for trend
**<30 nmol/L**	**30– < 50 nmol/L**	**50– < 75 nmol/L**	**75– < 100 nmol/L**	**≥100 nmol/L**
**Number**	3707	11104	16469	8843	3555	
**Crude model OR (95% CI)**	1.446 (0.758, 2.756)	1.762 (1.175, 2.641)	Ref	1.648 (1.106, 2.456)	1.489 (0.894, 2.480)	0.986
***P* value**	0.261	0.006		0.014	0.125	
**Model 1 OR (95% CI)**	1.973 (0.985, 3.952)	2.043 (1.331, 3.130)	Ref	1.280 (0.861, 1.902)	0.868 (0.519, 1.451)	0.014
***P* value**	0.055	0.001		0.220	0.586	
**Model 2 OR (95% CI)**	2.046 (1.011, 4.141)	2.105 (1.373, 3.228)	Ref	1.261 (0.851, 1.868)	0.852 (0.510, 1.424)	0.010
***P* value**	0.047	0.001		0.245	0.539	
**Model 3 OR (95% CI)**	2.038 (1.011, 4.109)	2.090 (1.361, 3.211)	Ref	1.260 (0.853, 1.862)	0.845 (0.502, 1.424)	0.011
***P* value**	0.047	0.001		0.244	0.525	

^a^The criteria for grouping serum 25(OH)D concentrations refer to the Institute of Medicine (IOM) and the Endocrine Society’s classification criteria for the five classifications, with the Institute of Medicine (IOM) considering 25(OH)D <  30 nmol/L to be vitamin D-deficient, 30– < 50 nmol/L to be vitamin D-insufficient, and ≥  50 nmol/L considered to be vitamin D-sufficient. According to the Endocrine Society, 25(OH)D < 50 nmol/L is vitamin D deficiency, 50– < 75 nmol/L is vitamin D insufficiency and 25(OH)D ≥ 75 nmol/L is vitamin D sufficiency.

Crude Model. Unadjusted.

Model 1. adjusted for Age, Race, Marital, Education, PIR.

Model 2. further adjusted for drinking habits, smoking habits based on Model 1.

Model 3. further adjusted for hypertension, dyslipidemia, calcium, total folate based on Model 2.

25(OH)D, 25-hydroxyvitamin D; OR, odds ratio; CI, confidence interval.

**Table 3:** VIF of the variables in three adjusted models.

Variable	Model 1	Model 2	Model 3
**25(OH)D**	1.139	1.249	1.322
**Gender**	1.075	1.232	1.286
**Age**	1.097	1.309	1.355
**Race**	1.128	1.199	1.306
**Marital**	1.354	1.599	1.613
**Education**	1.121	1.270	1.340
**PIR**	1.204	1.270	1.359
**BMI**		1.324	1.386
**Smoking habits**		1.521	1.737
**Drinking habits**		1.251	1.343
**Diabetes**			1.261
**Hypertension**			1.329
**Dyslipidemia**			1.724
**Calcium**			1.307
**Total folate**			1.440

VIF, Variance inflation factor; 25(OH)D, 25-hydroxyvitamin D; PIR, Poverty income ratio; BMI, body mass index.

**Fig 2:**
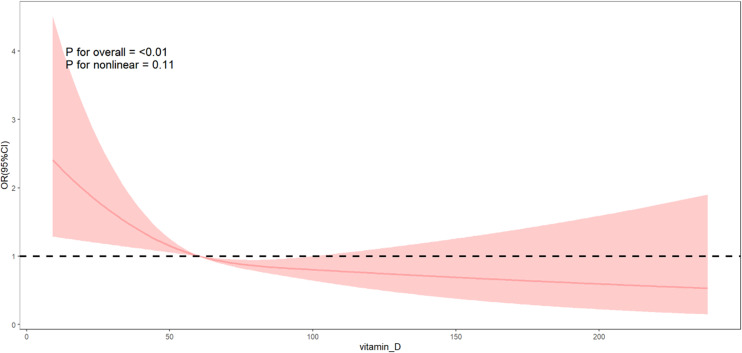
RCS method was used in the logistic regression to analyze the association between serum 25(OH)D concentration and the risk of CRC. OR, odds ratio; CI, confidence interval.

**Fig 3:**
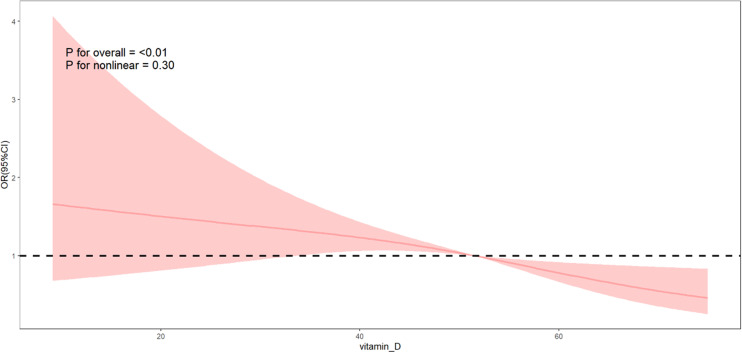
Restrict cubic spline method was used in the logistic regression to analyze the association between serum 25(OH)D concentration( ≤75nmol/L) and the risk of CRC. OR, odds ratio; CI, confidence interval.

The RCS (Fig 2) showed that a negative correlation between serum 25(OH)D concentration and the risk of CRC (*P* for overall < 0.01), and there was no non-linear trend between the two (*P* for nonlinearity = 0.11). We extracted data for participants with 25(OH)D ≤ 75 nmol/L, totaling 31,325 individuals, consisting of 190 with CRC and 31,135 without CRC, and performed logistic regression analysis. As shown in Table 4., we found that in three different models adjusted for potential confounding factors (Model 1, Model 2, Model 3), there was a significant linear inverse correlation between serum 25(OH)D concentration and the risk of CRC (*P* < 0.001). Additionally, the RCS (Fig 3) also supported this dose-response relationship.

**Table 4:** Association between serum 25(OH)D concentration(≤75nmol/L) and the risk of CRC in participants.

Variable	Crude model	Model 1	Model 2	Model 3
**OR (95% CI)**	0.985 (0.974, 0.996)	0.977 (0.964, 0.989)	0.976 (0.963, 0.989)	0.977 (0.964, 0.990)
***P* value**	0.0101	<0.001	<0.001	<0.001

Crude Model. Unadjusted

Model 1. Adjusted for age, race, marital, education, PIR.

Model 2. Further adjusted for drinking habits, smoking habits, based on Model 1.

Model 3. Further adjusted for hypertension, dyslipidemia, calcium, total folate based on Model 2.

OR, odds ratio; CI, confidence interval.

The results of subgroup analysis indicated that the relationship between serum 25(OH)D concentration and the risk of CRC was not consistent across different subgroups (Table 5). The relationship was statistically significant (*P* < 0.05) for the following subgroup analyses: males (OR = 0.988, 95%CI: 0.977–0.998), the 20–40 years age group (OR = 0.943, 95% CI: 0.902–0.986), participants aged > 60 years (OR = 0.992, 95% CI: 0.986–0.999), non-Hispanic whites (OR = 0.990, 95% CI: 0.983–0.997), participants with a BMI < 30 (OR = 0.989, 95% CI: 0.978–0.999), participants with a history of diabetes (OR = 0.985, 95% CI: 0.972–0.998), participants with a history of hypertension (OR = 0.992, 95% CI: 0.985–0.999), and participants with dyslipidemia (OR = 0.991, 95% CI: 0.983–0.999). These findings suggest that maintaining sufficient serum 25(OH)D levels may be particularly important for CRC risk reduction among individuals in these subgroups. However, we observed that the confidence intervals for some subgroup analyses were notably narrow (e.g., participants aged >60 years, non-Hispanic whites, and participants with metabolic conditions such as diabetes and hypertension). This suggests potential overfitting of the model, which may lead to an overestimation of precision in the effect estimates. Therefore, the interpretation of the subgroup analyses should be conducted with increased caution.

**Table 5:** Subgroup analysis and interaction test of serum 25(OH)D concentration and the risk of CRC.

Characteristic	OR (95% CI)	*P* value	*P*for interaction
**Gender**			0.235
Female	0.994 (0.987, 1.001)	0.067	
Male	0.988 (0.977, 0.998)	0.022	
**Age**			0.281
20–40	0.943 (0.902, 0.986)	0.010	
41–60	0.994 (0.978, 1.011)	0.510	
>60	0.992 (0.986, 0.999)	0.017	
**Race**			0.637
Mexican American	1.007 (0.985, 1.030)	0.515	
Non-Hispanic Black	0.999 (0.986, 1.012)	0.872	
Non-Hispanic White	0.990 (0.983, 0.997)	0.007	
Other	0.992 (0.972, 1.012)	0.418	
**BMI**			0.456
<30	0.989 (0.978, 0.999)	0.033	
≥30	0.993 (0.981, 1.001)	0.067	
**Diabetes**			0.217
Yes	0.985 (0.972, 0.998)	0.027	
No	0.994 (0.988, 1.001)	0.069	
**Hypertension**			0.795
Yes	0.992 (0.985, 0.999)	0.023	
No	0.992 (0.980, 1.003)	0.140	
**Dyslipidemia**			0.950
Yes	0.991 (0.983, 0.999)	0.032	
No	0.993 (0.985, 1.001)	0.092	

OR, odds ratio; CI, confidence interval; BMI, body mass index

## Discussion

This cross-sectional study used NHANES data from 2001 to 2018 to investigate the correlation between serum 25(OH)D concentration and the risk of CRC. The study concluded that there was a negative correlation between serum 25(OH)D concentration and the risk of CRC, and that lower serum vitamin D levels indicated a greater risk of CRC. However, when serum 25(OH)D concentrations were more adequate, the statistical significance of the negative association with CRC was no longer significant as serum 25(OH)D levels increased.

Previous clinical studies predominantly supported an inverse correlation between serum 25(OH)D levels and the risk of CRC, but some studies showed no statistically significant association. The discrepancies in these findings may stem from various factors, including differences in demographic characteristics such as geographic location and baseline vitamin D levels. Additionally, variations in measurement techniques of serum 25(OH)D concentration, errors in the processing or classification of 25(OH)D concentrations [23], small sample sizes, specific study populations, and potential confounding factors could all impact the results, limiting their generalizability and robustness. A prospective study of elderly men aged 70–88 years found no correlation between serum 25(OH)D concentrations and CRC risk, which could be due to their relatively low winter serum 25(OH)D concentrations (mean of 18 ng/ml) compared to the levels typically observed in younger and middle-aged individuals [24]. A large sample study conducted among Korean adults found that the inverse correlation between circulating 25(OH)D levels and CRC risk was weaker in individuals aged 50 years and older, compared to those younger than 50 [25]. Another cross-sectional study explored the relationship between vitamin D intake and CRC risk, but no significant association was found after adjusting for covariates [22]. This may be because vitamin D intake as an indicator was not as sensitive as serum 25(OH)D levels, which better reflected other factors in addition to intake, such as sun exposure and internal body conversion processes.

There is substantial evidence supporting an inverse relationship between serum 25(OH)D concentrations and the risk of CRC. However, the impact of increasing serum 25(OH)D levels on CRC risk may vary across different concentration ranges. A study indicated that within the observed range of vitamin D levels, there may be a specific concentration range of 25(OH)D that was significantly associated with the risk of developing CRC. The nested case-control study involving a multi-ethnic population demonstrated that, compared with participants in the lowest quintile of 25(OH)D levels (<16.8 ng/mL), those in all other quintiles experienced a reduction in CRC risk. Notably, participants in the top three quintiles (≥22.2 ng/mL) had significantly lower risks. The effect size for the fifth quintile showed a non-significant increase compared to the fourth quintile. The authors suggested a potential U-shaped dose-response relationship [13].Similarly, in a study conducted by Lee et al., CRC risk was reduced in participants within the second quartile of 25(OH)D concentrations (median: 22.3 ng/mL) compared to the lowest quartile (median: 15.7 ng/mL). However, participants in the higher quartiles (median: 26.7 ng/mL and 37.9 ng/mL) exhibited non-significant increases in CRC risk [12]. These findings imply that the benefit of vitamin D supplementation in reducing CRC risk may be greater among individuals with lower baseline serum 25(OH)D levels. Conversely, as serum 25(OH)D levels approach or exceed sufficiency, further increases in concentration may have diminishing or negligible effects on CRC risk reduction. In our study, compared to the 50–<75 nmol/L group, the two groups with serum 25(OH)D levels below 50 nmol/L demonstrated a statistically significant inverse association with CRC risk. However, for the two groups with serum 25(OH)D levels above 75 nmol/L, the effect sizes fluctuated and did not reach statistical significance. This suggests that serum 25(OH)D levels may, once exceeding a certain threshold, help maintain CRC risk at a relatively low level.

Many organizations and guidelines base their recommendations for optimal vitamin D levels primarily on its general health benefits and its role in maintaining musculoskeletal health. The main sources of vitamin D include sunlight exposure, dietary supplements, and food. However, factors such as skin pigmentation and the use of sunscreen can significantly reduce the synthesis of vitamin D from sunlight [26]. Furthermore, only a few foods, such as oily fish, fatty fish and certain types of mushrooms, naturally contain high levels of vitamin D. To address this, countries like the United States and Canada have implemented food fortification programs, adding vitamin D to various foods to supplement daily diets [19,27]. Despite these measures, there remains substantial variation among organizations regarding the recommended standards for serum vitamin D concentrations. The Scientific Advisory Committee on Nutrition in the UK suggests that a serum 25(OH)D concentration of at least 25 nmol/L is necessary to reduce the risk of rickets and osteomalacia [17]. IOM in the United States proposes that a serum 25(OH)D concentration of 40 nmol/L can meet the skeletal health needs of about half of the population, and a level above 50 nmol/L can meet the needs of 97.5% of the population [15]. The Endocrine Society’s Vitamin D Working Group believes that a serum 25(OH)D concentration below 50 nmol/L should be considered vitamin D deficiency, and a level above 75 nmol/L is considered adequate vitamin D status, which helps to maximize the positive effects of vitamin D on calcium, bone, and muscle metabolism [16]. Additionally, some studies have suggested that to reduce the risk of diseases beyond skeletal health, serum vitamin D levels should be maintained above 50 nmol/L [28]. In the context of reducing the risk of CRC, several studies have proposed alternative recommendations for serum 25(OH)D levels and vitamin D intake. A meta-analysis of 15 nested case-control studies or cohort studies from 14 countries showed that, compared to a concentration of 5 ng/mL, a 25(OH)D level of 50 ng/mL was associated with a 60% reduction in the risk of CRC, and 30 ng/mL was associated with a 33% reduction in the risk of CRC. It is suggested that the ideal target 25(OH)D concentration for preventing CRC should be ≥ 50 ng/mL [23]. Another systematic review supported the negative correlation between 25(OH)D and CRC risk and recommended maintaining 25(OH)D levels at least 75 nmol/L [29]. Some studies suggest that the inverse relationship between vitamin D and CRC is more pronounced in colon cancer than in rectal cancer, and the recommended daily intake of vitamin D, ranging from 200 to 600 IU, may be too low to effectively reduce the risk of developing CRC [30].The negative correlation presented by the RCS plots for the full concentration of 25(OH)D in Fig 2 of the results of this study can be observed in the trend of the effect value. Further multivariate logistic regression and restricted cubic spline plots for serum 25(OH)D concentrations less than or equal to 75 nmol/L showed a significant negative linear correlation. Specifically, for each 1 nmol/L increase in serum 25(OH)D concentration, the risk of CRC is reduced by approximately 2.3% (95% CI: 0.964–0.990, *P* < 0.001). In conjunction with guidelines from various organizations regarding serum vitamin D levels, our findings support the notion that a serum vitamin D concentration level greater than 75 nmol/L may play a significant role in maintaining a low risk of CRC.

The specific mechanisms of the inverse association between vitamin D and the risk of CRC are likely related to the following aspects: In the study by Vaughan-Shaw et al., the link between vitamin D deficiency and the risk of CRC was revealed using patient-derived epithelial organoids (ex vivo) and CRC cell lines (in vitro). The study found that the active form of vitamin D, 1,25-dihydroxyvitamin D [1,25(OH)2D], could upregulate the expression of the CRC tumor suppressor gene CDH1, and lead to significant changes in the expression of six other genes associated with cancer occurrence. Through gene ontology analysis, researchers found that 1,25(OH)2D could regulate biological processes related to cancer development, such as the Wnt signaling pathway and cell death. This study suggests that it provides a mechanistic explanation for the potential causal relationship between vitamin D and CRC [31]. In addition, 1,25(OH)2D inhibits the Wnt/β-catenin signaling pathway through various mechanisms, which is key to the occurrence and development of colon cancer [32–34]. Calcitriol has a broad immunomodulatory effect on various immune cells, which may help it combat CRC [35]. 1,25(OH)2D is involved in detoxification in the gut and improvement of the intestinal microenvironment, affecting the occurrence and development of CRC by controlling the expression of antioxidant phase I and II enzymes, and participating in the metabolism of steroids, bile acids, xenobiotics, and other compounds [36]. Therefore, there are also studies that suggest vitamin D deficiency may be a consequence of the development of CRC, rather than its cause [37]. On the other hand, the results of several Mendelian randomization (MR) studies targeting different populations did not support a causal relationship between circulating vitamin D levels and the risk of CRC [38–41]. These discrepancies may arise from several factors. MR studies use genetic variants as proxies for exposure, minimizing confounding and reverse causation but potentially overlooking the complexity of vitamin D metabolism and its interaction with environmental factors. MR studies focus on lifetime exposure to genetically determined vitamin D levels, which may not reflect transient changes due to diet or sun exposure. Additionally, genetic variants associated with vitamin D levels might influence other biological pathways, introducing pleiotropy and complicating causal inference. In contrast, observational studies are more susceptible to confounding factors like lifestyle and comorbidities, which may inflate observed associations. Reconciling these differences requires integrated analyses combining genetic, environmental, and experimental data to better understand the role of vitamin D in CRC prevention.

This study was based on data from the NHANES database for 9 cycles from 2001–2018, with a total of 43,678 participants included, which allows for generalizability to the population observed in this study and exploring further studies on other factors more commonly associated with vitamin D and CRC. The serum 25(OH)D concentrations included here as an exposure factor is more stable for vitamin D targeted at dietary intake or supplementation. We did not use the conventional four-category classification for the study, but grouped the vitamin D levels provided by the different guidelines, which are different, and chose the critical interval of vitamin D levels as a dummy variable to make the study more clinically relevant and thus obtain more socially relevant study conclusions. We included factors related to demographics, lifestyle, history of other diseases, and dietary intake as covariates to make the results of the study more reliable, and we performed subgroup analyses and interaction tests to investigate the correlation between the two in different populations. At the same time, this study has its limitations. As a cross-sectional study, this study includes serum 25(OH)D concentrations and CRC incidence at the same time, which can only investigate the correlation, not the causal relationship between the two. Although several covariates were included for adjustment, there may still be unmeasured potential confounding factors that could affect the interpretation of the findings. The NHANES data contained participants’ self-reported eating, smoking, and drinking habits, and although measures were taken during data collection to reduce recall bias and improve accuracy, reported behaviors may not be consistent with reality, affecting the reliability of the study. Serum 25(OH)D concentrations may be measured using different techniques in different studies, including liquid chromatography-tandem mass spectrometry, chemiluminescence immunoassay, etc., and methodological differences may lead to reduced comparability between study results. Concerns have been raised regarding the narrow confidence intervals in subgroup analyses, which may have led to an overestimation of precision in effect estimates. This issue is particularly relevant in subgroups with smaller sample sizes, where variability is inherently greater. Consequently, the statistical significance observed in certain subgroup analyses may not fully reflect the true relationship between serum 25(OH)D concentrations and CRC risk. Future studies with larger sample sizes and independent validation cohorts are needed to confirm these findings and enhance their robustness. Given that vitamin D levels are influenced by race, age, geographic location, sunlight exposure, and diet, future research should focus on diverse populations to better understand the role of vitamin D in CRC prevention across different subgroups. Longitudinal or interventional study designs should also be employed to more rigorously assess the causal relationship between vitamin D and CRC risk. From a public health and clinical perspective, improving vitamin D status may be a cost-effective strategy for CRC prevention. Routine screening of serum 25(OH)D levels in high-risk populations—such as older adults, individuals with limited sun exposure, and those with metabolic disorders—could facilitate early identification and intervention for vitamin D deficiency. In clinical practice, integrating vitamin D assessment into regular health check-ups for CRC high-risk individuals, including those with a family history, inflammatory bowel disease, or obesity, may enhance early prevention efforts. Targeted supplementation should be considered for individuals with serum 25(OH)D levels below 75 nmol/L, which our study suggests may be beneficial for CRC risk reduction. Public health initiatives should focus on promoting safe sunlight exposure, increasing dietary intake through food fortification programs, and encouraging appropriate vitamin D supplementation among at-risk populations. Future research should continue to refine optimal vitamin D targets for CRC prevention and evaluate the effectiveness of these interventions across diverse populations, providing stronger evidence to support public health policies and preventive clinical strategies.

## Conclusion

In conclusion, this study found that serum 25(OH)D concentration was negatively correlated with the risk of CRC. The linear trend between the two was more significant at 25(OH)D ≤ 75 nmol/L, with 25(OH)D deficiency and insufficiency leading to an increased risk of CRC. To reduce the risk of CRC, the findings of this study recommend maintaining 25(OH)D levels at a minimum of 75 nmol/L, which may help in keeping the risk of CRC at a lower level. This study highlights the importance of appropriate vitamin D supplementation and maintaining reasonable control of serum 25(OH)D levels in the prevention of CRC. However, further evidence from large-scale prospective clinical studies is still needed.
